# Dynamic Nutritional Decline During the Diagnostic-to-Treatment Interval Is Associated with Treatment Resilience and Survival in Unresectable Pancreatic Ductal Adenocarcinoma

**DOI:** 10.3390/nu18121998

**Published:** 2026-06-19

**Authors:** Nobuhiko Shinohara, Shinji Oe, Koichiro Miyagawa, Yuichi Honma, Kenta Kajitani, Tsuyoshi Ueda, Noriyoshi Ogino, Shinsuke Kumei, Tatsuyuki Watanabe, Michihiko Shibata, Masaru Harada

**Affiliations:** Third Department of Internal Medicine, School of Medicine, University of Occupational and Environmental Health, 1-1 Iseigaoka, Yahatanishi-ku, Kitakyushu 807-8555, Japan; n-shino9144@med.uoeh-u.ac.jp (N.S.); koichiro@med.uoeh-u.ac.jp (K.M.); y-homma@med.uoeh-u.ac.jp (Y.H.); k.kajitani1115@gmail.com (K.K.); jenmajayro0930@gmail.com (T.U.); monkmondays@gmail.com (N.O.); msrharada@med.uoeh-u.ac.jp (M.H.)

**Keywords:** pancreatic cancer, geriatric nutritional risk index, chemotherapy tolerability, nutritional decline, nutritional vulnerability, cancer cachexia, treatment resilience, diagnostic-to-treatment interval

## Abstract

**Background/Objectives:** Patients with unresectable pancreatic ductal adenocarcinoma (UR-PDAC) are vulnerable to rapid nutritional deterioration. The clinical relevance of short-term nutritional change during the diagnostic-to-treatment interval (DTI) remains unclear. In this study, we evaluated whether a dynamic change in the Geriatric Nutritional Risk Index (ΔGNRI) during the DTI is associated with treatment tolerability, treatment continuity, and survival. **Methods:** This single-center retrospective study included 120 patients with histologically confirmed UR-PDAC who initiated first-line palliative chemotherapy between January 2016 and April 2024. ΔGNRI was defined as the GNRI immediately before chemotherapy minus the GNRI at the initial visit. ΔGNRI was primarily analyzed as a continuous variable, and an exploratory cut-off value of −6.8 was determined by receiver operating characteristic analysis. One-to-one propensity score matching was performed as a sensitivity analysis. Clinically significant adverse events (AEs) were defined as grade ≥3 AEs or AEs requiring treatment modification, hospitalization, or treatment discontinuation. **Results:** Patients in the GNRI-decreased group had more frequent clinically significant non-hematologic AEs, including gastrointestinal AEs, higher hospitalization rates due to AEs, and more frequent early treatment discontinuation. ΔGNRI remained independently associated with early treatment discontinuation and failure to transition to second-line therapy in multivariable analyses. Patients in the GNRI-decreased group also had significantly shorter times to treatment failure and overall survival. These findings were consistent in propensity score-matched analyses. **Conclusions:** Dynamic nutritional decline during the DTI was associated with impaired treatment resilience and poor survival outcomes in UR-PDAC. ΔGNRI may help identify patients with emerging nutritional vulnerability before chemotherapy.

## 1. Introduction

Patients with unresectable pancreatic ductal adenocarcinoma (UR-PDAC) frequently experience nutritional and metabolic deterioration even before chemotherapy initiation. These changes may reduce physiologic reserve, impair treatment resilience, and contribute to adverse clinical outcomes. Combination chemotherapy regimens such as gemcitabine plus nab-paclitaxel (GnP) and FOLFIRINOX have improved survival outcomes in patients with UR-PDAC [1,2]. However, these regimens are frequently accompanied by substantial adverse events (AEs), and maintaining treatment continuity is often challenging in routine practice. In particular, early treatment discontinuation of first-line chemotherapy and failure to proceed to second-line therapy are clinically important events that may reflect limited treatment tolerability and reduced physiologic reserve [3,4]. Therefore, there is a need for practical markers that can identify patients at risk of treatment intolerance before chemotherapy initiation to support timely multidisciplinary supportive care.

Patients with UR-PDAC are prone to early nutritional deterioration due to multiple factors, including pancreatic exocrine insufficiency, biliary obstruction, anorexia, systemic inflammation, and cancer-related catabolism [5,6]. Cancer cachexia, driven by tumor-induced metabolic derangement, can reduce physiologic reserve and increase vulnerability to chemotherapy-related toxicity [7,8,9]. As a result, dose reduction, treatment interruption, hospitalization, or early discontinuation of treatments may occur, potentially disrupting treatment sequencing, including the transition to second-line therapy. Nutritional assessment may provide an important clinical framework for estimating treatment resilience in UR-PDAC.

Among nutritional indices, the Geriatric Nutritional Risk Index (GNRI) is an objective and simple measure calculated from serum albumin and body weight (BW). Recent studies and meta-analyses have supported the clinical relevance of GNRI in pancreatic cancer, showing that a low GNRI is associated with poor overall survival and may also predict postoperative complications, including postoperative pancreatic fistula [10,11,12]. A recent systematic review of nutritional screening and assessment tools in pancreatic neoplasms further suggested that poor nutritional status as assessed using tools such as GNRI, the Prognostic Nutritional Index, the Controlling Nutritional Status score, Nutritional Risk Screening 2002, and the modified Glasgow Prognostic Score predicts all-cause mortality while emphasizing that the optimal tool may depend on the clinical setting and purpose of assessment [13]. However, most previous studies have relied on static single-time-point assessments, such as at diagnosis, before surgery, after surgery, or at treatment initiation. Therefore, these approaches may fail to capture rapid physiologic deterioration occurring before treatment initiation. In routine clinical practice, the diagnostic-to-treatment interval (DTI), defined as the interval from the initial visit to the initiation of the first-line chemotherapy, includes invasive diagnostic procedures, biliary interventions, symptom management, nutritional assessment, and other supportive care. This interval may function as a clinically informative stress period during which latent vulnerability becomes apparent.

Although the chronological duration of the DTI alone may not necessarily determine treatment outcomes [14], the magnitude of nutritional deterioration during this interval may provide additional clinically relevant information. We hypothesized that a rapid decline in GNRI during the DTI would identify patients with limited treatment resilience, even when their nutritional status appeared to be preserved at the initial visit.

In this study, we investigated the clinical significance of dynamic changes in GNRI (ΔGNRI) from the initial visit to immediately before first-line chemotherapy in patients with UR-PDAC receiving palliative chemotherapy. Specifically, we evaluated whether ΔGNRI was associated with early discontinuation of first-line therapy, failure to transition to second-line therapy, and survival outcomes, with the aim of clarifying its potential role as a practical marker of pre-treatment physiologic vulnerability relevant to supportive care planning.

## 2. Materials and Methods

### 2.1. Study Design and Patients

**Figure 1:**
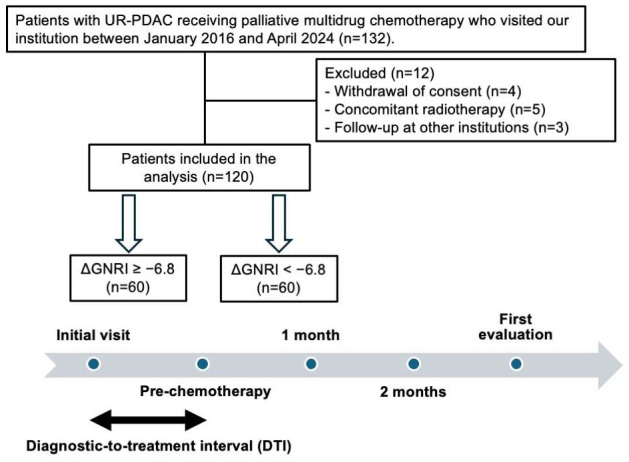
Study design, timeline, and patient selection.

This was a single-center retrospective observational study. We included patients who were histologically diagnosed with UR-PDAC and initiated palliative multi-agent chemotherapy, consisting of GnP or modified FOLFIRINOX (mFOLFIRINOX), at our institution between January 2016 and April 2024. Of the 132 patients initially identified, 12 were excluded due to withdrawal of consent for reasons unrelated to AEs (*n* = 4), concomitant radiotherapy (*n* = 5), or loss to follow-up due to transfer for continued chemotherapy at another institution (*n* = 3). The final study cohort consisted of 120 patients (Figure 1). The patients were followed from the date of their initial visit to our institution until death or December 2025, whichever came first.

### 2.2. Diagnostic-to-Treatment Interval

The DTI was defined as the interval between the first visit to our institution for suspected or confirmed pancreatic cancer and the initiation of first-line chemotherapy. This interval included diagnostic imaging, pathological confirmation, biliary interventions when indicated, symptom management, nutrition assessment, and other supportive care before chemotherapy initiation.

### 2.3. Clinical Data Collection

Clinical data were extracted from electronic medical records, including age, sex, Eastern Cooperative Oncology Group performance status (ECOG PS), anthropometric measurements, laboratory findings, tumor-related factors, treatment characteristics, adverse events, information on pancreatic enzyme replacement therapy (PERT), opioid use, dietary counseling during the DTI, and clinical outcomes.

The tumor-related factors included TNM stage according to the 8th edition of the TNM classification, tumor location, distant metastasis, metastatic site, ascites, and biliary drainage during the DTI. Data were systematically collected at the following time points: initial visit, immediately before first-line chemotherapy, 1 and 2 months after chemotherapy initiation, and at the first radiologic response assessment.

### 2.4. Nutritional, Inflammatory, and Muscle Indices

Inflammatory status was assessed using the neutrophil-to-lymphocyte ratio (NLR) and modified Glasgow Prognostic Score (mGPS). The mGPS was calculated using serum C-reactive protein and albumin levels according to established criteria [15]. Skeletal muscle mass was evaluated using the psoas muscle index (PMI), which was assessed using diagnostic abdominal computed tomography (CT) images obtained as part of the initial diagnostic work-up. The cross-sectional areas of the right and left psoas muscles were manually traced on axial images at the level of the third lumbar vertebra and summed. The PMI was calculated by normalizing the bilateral psoas muscle area to the square of the patient’s height (cm^2^/m^2^). Low skeletal muscle mass was defined as <6.36 cm^2^/m^2^ for men and <3.92 cm^2^/m^2^ for women, according to previously reported Japanese reference values [16].

### 2.5. Definition of GNRI and ΔGNRI

The GNRI was calculated as follows: GNRI = [14.89 × serum albumin (g/dL)] + [41.7 × (actual BW/ideal BW)]. Ideal BW was calculated as height (m) × height (m) × 22. When the actual BW exceeded the ideal BW, the BW ratio was capped at 1.0 according to the original method [17].

ΔGNRI was defined as the GNRI immediately before first-line chemotherapy (pre-chemotherapy GNRI) minus the GNRI at the initial visit (initial-visit GNRI). Accordingly, a negative ΔGNRI value indicated deterioration in nutritional status during the DTI. ΔGNRI was primarily analyzed as a continuous variable. For descriptive comparisons and Kaplan–Meier analyses, patients were additionally categorized using an exploratory cut-off value of −6.8, determined by receiver operating characteristic (ROC) curve analysis for early treatment discontinuation using the Youden index (sensitivity, 57.8%; specificity, 94.4%; area under the curve (AUC), 0.79; 95% confidence interval (CI), 0.69–0.89; Supplementary Figure S1). Patients were categorized into the GNRI-maintained group (ΔGNRI ≥ −6.8) and the GNRI-decreased group (ΔGNRI < −6.8). Because this threshold was derived from the present single-center cohort, it was used for exploratory group-based analyses, whereas multivariable models also evaluated ΔGNRI as a continuous variable.

### 2.6. Endpoints

The primary endpoints were early treatment discontinuation and failure to transition to second-line therapy. Early treatment discontinuation was defined as discontinuation of first-line chemotherapy before the first radiologic response evaluation. Transition to second-line therapy was defined as the initiation of second-line treatment after discontinuation of the first-line therapy.

The secondary endpoints were time to treatment failure (TTF) and overall survival (OS). TTF was defined as the interval from chemotherapy initiation to treatment discontinuation, disease progression, or death, whichever occurred first. OS was defined as the interval from chemotherapy initiation to death from any cause or last follow-up.

AEs were assessed based on medical records and categorized with reference to the Common Terminology Criteria for Adverse Events (CTCAE), version 5.0 [18]. In this study, clinically significant AEs were defined as grade ≥3 AEs or AEs requiring dose reduction, treatment interruption, hospitalization, or permanent discontinuation of first-line chemotherapy. Hematologic AEs were tabulated as grade ≥3 events according to CTCAE version 5.0 based on laboratory data. Non-hematologic AEs included all clinically significant AEs other than hematologic AEs. Gastrointestinal AEs were defined as clinically significant AEs categorized under “Gastrointestinal disorders” in CTCAE and were analyzed as a grouped category rather than as individual symptoms. Infectious AEs included clinically significant infections, including cholangitis. Other non-hematologic AEs, such as fatigue, peripheral neuropathy, and interstitial pneumonitis, were included in the non-hematologic category but were not separately tabulated. Early AEs were defined as clinically significant AEs occurring before the first radiologic response evaluation.

### 2.7. Statistical Analysis

Continuous variables were compared using Student’s t-test or the Mann–Whitney U test, as appropriate, and categorical variables using the chi-square test or Fisher’s exact test. A two-way repeated-measures analysis of variance (ANOVA) was performed to evaluate the interaction between time and ΔGNRI group. When the assumption of sphericity was violated, Greenhouse–Geisser correction was applied.

Multivariable logistic regression analysis was used to identify factors associated with early treatment discontinuation and failure to transition to second-line therapy. Cox proportional hazards models were used to evaluate associations with TTF and OS. Changes in GNRI were analyzed as a continuous variable in the primary multivariable analyses to preserve statistical power and avoid overreliance on a data-derived cut-off value. Covariates were selected based on clinical relevance and the number of events. To reduce multicollinearity related to biliary obstruction, total bilirubin was used in multivariable models.

To minimize confounding, propensity score matching was performed as a sensitivity analysis. Propensity scores were estimated using a logistic regression model incorporating clinically relevant covariates potentially associated with nutritional status or treatment outcomes, including serum carbohydrate antigen 19-9 (CA19-9), tumor location, distant metastasis, liver metastasis, mGPS, ascites, chemotherapy regimen, ECOG PS, and DTI. Patients were matched 1:1 using nearest-neighbor matching without replacement, with a caliper width of 0.2 of the standard deviation of the logit of the propensity score. Covariate balance after matching was assessed using standardized mean differences (SMDs), and an SMD <0.20 was considered indicative of acceptable balance [19].

Cases with missing values were handled using a complete-case approach. Statistical analyses were performed using EZR version 1.61 (Saitama Medical Center, Jichi Medical University, Saitama, Japan) [20]. A two-sided *p* value < 0.05 was considered statistically significant.

## 3. Results

### 3.1. Patient Characteristics

The patient selection flow is shown in Figure 1. ROC curve analysis for early treatment discontinuation identified an exploratory ΔGNRI cut-off value of −6.8. The final cohort consisted of 120 patients, including 60 in the GNRI-maintained group (ΔGNRI ≥ −6.8) and 60 in the GNRI-decreased group (ΔGNRI < −6.8). The baseline characteristics of the overall cohort are presented in Table 1, and more detailed baseline data are provided in Supplementary Table S1. To provide additional information regarding the patient selection process, the baseline characteristics of the 12 excluded patients are summarized in Supplementary Table S2. Given that the groups were defined according to the ΔGNRI, the ΔGNRI and pre-chemotherapy GNRI differed significantly between the two groups (*p* < 0.001). Serum CA19-9 levels were also significantly higher in the GNRI-decreased group (*p* = 0.003). The DTIs were similar between the two groups. The median DTI was 0.8 months (interquartile range, 0.5–1.3; range, 0.2–3.9). The use of PERT and dietary counseling during the DTI did not differ significantly between the groups. Opioid use during the DTI was numerically more frequent in the GNRI-decreased group, although the difference was not statistically significant.

To assess the impact of confounding, propensity score matching was performed as a sensitivity analysis, and the baseline characteristics of the propensity score-matched cohort are shown in Supplementary Table S3. After matching, most major covariates were well balanced; however, minor residual imbalance was observed for distant metastasis, chemotherapy regimen, biliary drainage during the DTI, and total bilirubin (SMD slightly > 0.20). To account for the clinical impact of biliary obstruction, total bilirubin was additionally included in the multivariable models.

Patients with unresectable pancreatic ductal adenocarcinoma (UR-PDAC) who initiated palliative multi-agent chemotherapy at our institution between January 2016 and April 2024 were retrospectively identified (*n* = 132). After excluding patients who withdrew consent for reasons unrelated to adverse events (*n* = 4), received concomitant radiotherapy (*n* = 5), or continued treatment at other institutions with unavailable outcome ascertainment (*n* = 3), a total of 120 patients were included in the final analysis. The diagnostic-to-treatment interval (DTI) was defined as the interval from the initial visit to the initiation of first-line chemotherapy. The GNRI was assessed at the initial visit and immediately before chemotherapy initiation, and the ΔGNRI was calculated during this interval. Patients were stratified using the exploratory cut-off value of −6.8 into a GNRI-maintained group (ΔGNRI ≥ −6.8, *n* = 60) and a GNRI-decreased group (ΔGNRI < −6.8, *n* = 60). First evaluation refers to the first radiologic response assessment after chemotherapy initiation. Abbreviations: UR-PDAC, unresectable pancreatic ductal adenocarcinoma; GNRI, Geriatric Nutritional Risk Index; ΔGNRI, change in Geriatric Nutritional Risk Index.

### 3.2. Nutritional Trajectories During the DTI

Longitudinal changes in BW, serum albumin, and GNRI from the initial visit to the early treatment period are shown in Supplementary Table S4 and Supplementary Figure S2. Overall, the GNRI declined from the initial visit to immediately before chemotherapy initiation.

Importantly, the magnitude of the GNRI decline differed significantly between the two groups. A two-way repeated-measures ANOVA showed a significant group × time interaction for the GNRI (Greenhouse–Geisser corrected *p* < 0.001), indicating a steeper decline during the DTI in the GNRI-decreased group. Similar group × time interactions were observed for body weight (*p* = 0.028) and serum albumin (*p* < 0.001) (Supplementary Table S4), indicating that the more pronounced GNRI decline in the GNRI-decreased group was accompanied by significant reductions in its core components. Based on this observation, the ΔGNRI was used as the primary exposure variable in subsequent analyses.

### 3.3. Treatment Tolerability and Early Treatment Discontinuation

Treatment-related clinically significant AEs and treatment discontinuation according to ΔGNRI status are shown in Table 2. The GNRI-decreased group experienced a higher frequency of AEs, including gastrointestinal AEs. Hospitalization due to AEs was also more frequent in this group.

Early treatment discontinuation (before the first radiologic assessment) occurred significantly more often in the GNRI-decreased group, and early discontinuation due to AEs was observed exclusively in this group. In multivariable logistic regression analysis, the ΔGNRI (as a continuous variable) remained independently associated with early treatment discontinuation (Table 3). This association was also observed in the propensity score–matched cohort, in which early treatment discontinuation was more frequent in the GNRI-decreased group than in the GNRI-maintained group (43.8% vs. 9.4%, *p* = 0.004; Supplementary Table S5), and remained robust across sequentially adjusted sensitivity models (Supplementary Table S6).

### 3.4. Transition to Second-Line Chemotherapy

The rate of transition to second-line therapy was significantly lower in the GNRI-decreased group than in the GNRI-maintained group (Table 2). In multivariable logistic regression analysis, the ΔGNRI remained independently associated with failure to transition to second-line chemotherapy (Table 3). In the propensity score-matched cohort, failure to transition to second-line therapy was more frequent in the GNRI-decreased group than in the GNRI-maintained group (75.0% vs. 28.1%, *p* < 0.001; Supplementary Table S5). This association remained directionally consistent in sensitivity models incorporating additional covariates and categorical ΔGNRI status (Supplementary Table S7).

### 3.5. Survival Outcomes

**Figure 2:**
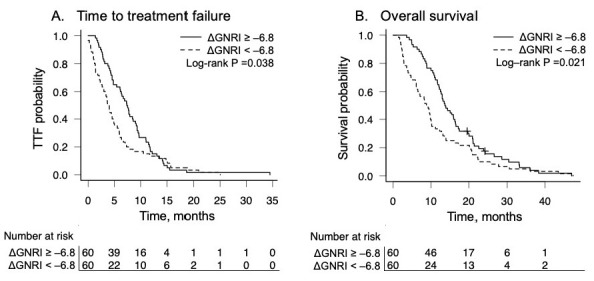
Kaplan–Meier analyses for time to treatment failure and overall survival according to ΔGNRI status. Kaplan–Meier curves comparing (**A**) time to treatment failure (TTF) and (**B**) overall survival (OS) between patients with ΔGNRI ≥ −6.8 and those with ΔGNRI < −6.8 during the diagnostic-to-treatment interval. Median TTF was 7.5 months (95% CI, 5.4–9.0) in the ΔGNRI ≥ −6.8 group and 3.9 months (95% CI, 2.7–4.9) in the ΔGNRI < −6.8 group. Median OS was 13.8 months (95% CI, 12.1–16.5) in the ΔGNRI ≥ −6.8 group and 9.0 months (95% CI, 6.1–10.2) in the ΔGNRI < −6.8 group. Survival distributions were compared using the log-rank test. Numbers at risk are shown beneath each plot. Abbreviations: CI, confidence interval; GNRI, Geriatric Nutritional Risk Index; ΔGNRI, change in Geriatric Nutritional Risk Index; OS, overall survival; TTF, time to treatment failure, +: censored observations, including patients who were alive at the last follow-up.

Kaplan–Meier curves for TTF and OS according to ΔGNRI status are shown in Figure 2. Patients in the GNRI-decreased group had significantly shorter TTFs and OS (log-rank *p* < 0.05 for both). In multivariable Cox proportional hazards models, the ΔGNRI remained independently associated with both TTF and OS after adjustment for covariates such as ECOG PS and chemotherapy regimen (Table 4). Similar associations were observed in sensitivity analyses incorporating additional covariates (Supplementary Table S8A,B) and in the PS-matched cohort (Supplementary Figure S3). In exploratory Cox interaction analyses using the median DTI as the cut-off, there was no clear evidence that DTI duration significantly modified the association between the ΔGNRI as a continuous variable and survival outcomes (P for interaction = 0.110 for TTF and 0.695 for OS; Supplementary Table S9).

### 3.6. Comparison Between Dynamic and Static Nutritional Assessments

To compare the clinical relevance of the dynamic nutritional indicator (ΔGNRI) with the static pre-chemotherapy GNRI (using the conventional threshold), we performed a multivariable analysis for early treatment discontinuation in which both variables were entered simultaneously into the same model. In that model, the ΔGNRI remained independently associated with the outcome, whereas the static pre-chemotherapy GNRI lost statistical significance (Supplementary Table S10).

### 3.7. Sensitivity Analyses Incorporating Inflammatory Dynamics and Biliary Obstruction

To assess whether the association between the ΔGNRI and early treatment discontinuation could be explained by inflammatory dynamics or biliary obstruction, additional models including changes in NLR (ΔNLR) and total bilirubin were examined (Supplementary Table S11). The ΔGNRI remained significantly associated with early treatment discontinuation across models. In contrast, the ΔNLR showed only a borderline association when assessed alone, and this association was attenuated when the ΔGNRI was included in the same model. Further adjustment for total bilirubin did not materially alter the association between the ΔGNRI and the outcome, and neither the ΔNLR nor bilirubin remained independently significant. In addition, the correlation between the ΔGNRI and ΔNLR was weak, indicating no strong collinearity (Supplementary Figure S4).

## 4. Discussion

In this study, we found that dynamic nutritional decline, assessed by the ΔGNRI during the DTI, was consistently associated with treatment resilience, treatment continuity, and survival outcomes in patients with UR-PDAC. The main finding was that short-term deterioration in GNRI before chemotherapy initiation identified patients at high risk of treatment intolerance, including non-hematologic AEs, gastrointestinal AEs, hospitalization due to adverse events, and early discontinuation of first-line chemotherapy. Notably, the ΔGNRI predicted clinically meaningful outcomes more strongly than the absolute GNRI value measured immediately before chemotherapy initiation. Even when the ΔGNRI and the pre-chemotherapy GNRI were entered simultaneously into the same model, only the ΔGNRI remained independently associated with outcomes. These findings suggest that the direction and velocity of nutritional decline, which are not captured by static single-time-point assessment, may more sensitively reflect underlying treatment vulnerability.

The clinical relevance of this dynamic assessment was particularly evident in treatment tolerability. Patients in the GNRI-decreased group experienced more clinically significant non-hematologic AEs, including gastrointestinal AEs, and were more likely to discontinue treatment early. These findings are consistent with the interpretation that nutritional decline during the DTI reflects the depletion of systemic reserve, thereby increasing susceptibility to chemotherapy-related toxicity [21,22]. By contrast, grade ≥3 hematologic toxicities were less clearly differentiated between groups. This suggests that the ΔGNRI may reflect functional treatment tolerance and host reserve rather than simply overall treatment exposure or myelosuppressive toxicity.

Reduced treatment tolerability directly affects treatment sequencing. In our cohort, the rate of transition to second-line chemotherapy was lower in the GNRI-decreased group. In UR-PDAC, maintaining treatment intensity and delivering sequential lines of therapy are important determinants of prognosis [3,4]. Therefore, physiologic decline before treatment initiation may have meaningful downstream consequences for long-term outcomes. In this context, the ΔGNRI should not be viewed solely as a survival predictor, but rather as a practical marker of pre-treatment vulnerability linked to treatment intolerance, impaired treatment continuity, and subsequent survival outcomes. Nutritional decline during the DTI should not be dismissed as a transient or clinically minor change, even when nutritional status appears preserved at the initial visit.

We next considered the biological processes reflected by the ΔGNRI. In the present study, the correlation between the ΔGNRI and the dynamic inflammatory marker ΔNLR was weak. Moreover, the association between the ΔGNRI and clinical outcomes remained robust even after adjustment for the ΔNLR and biliary obstruction. These findings suggest that the ΔGNRI captures a broader dimension of host vulnerability that cannot be explained by inflammatory fluctuation alone. This may include a composite effect of reduced metabolic reserve, progressive catabolism, procedural burden during the diagnostic workup, psychological stress, and tumor–host metabolic interaction [23,24]. Accordingly, ΔGNRI may be better understood not as a surrogate for a single biological mechanism, but as a composite clinical signal reflecting latent treatment vulnerability arising from the interaction between tumor biology, host metabolic reserve, and clinical stress during the pre-treatment period.

To further address potential confounding by tumor burden and disease distribution, we performed propensity score matching incorporating serum CA19-9, tumor location, and metastatic status. The persistence of the association between the ΔGNRI and outcomes in the matched cohort indicates that the observed relationship is unlikely to be explained solely by imbalance in baseline clinical factors. Nevertheless, residual imbalance remained in some disease- and treatment-related variables, and the propensity score-matched findings should be interpreted as supportive rather than definitive.

A key conceptual implication of this study is that the DTI should not be regarded as a passive waiting period, but rather as a biologically active interval during which clinically meaningful physiologic decline may occur. This interpretation is consistent with contemporary models of multiphasic prehabilitation, which conceptualize the pre-treatment period as a window of physiologic vulnerability and modifiability across the cancer continuum [25]. Although the chronological length of the DTI itself may not necessarily determine prognosis, our findings suggest that the biological intensity of decline during this interval may be more relevant. In exploratory interaction analyses, there was no clear evidence that DTI duration significantly modified the association between the ΔGNRI and survival outcomes. These results suggest that the magnitude of nutritional decline during the DTI may be more clinically informative than the duration of the interval alone. However, because this analysis was exploratory and limited by sample size, further studies are needed to clarify whether DTI duration modifies the clinical impact of nutritional deterioration.

Rapid GNRI decline may reflect active catabolic processes consistent with the early phases of cancer cachexia [8,9,26]. Cancer cachexia is a multifactorial syndrome characterized by reduced nutritional intake, systemic inflammation, metabolic dysregulation, and progressive loss of body weight and skeletal muscle mass. Although the ΔGNRI does not directly diagnose cachexia, it may capture an early cachexia-related clinical trajectory during the DTI, particularly when nutritional deterioration occurs over a short period before chemotherapy initiation. In this context, the DTI may function as a physiologic stress period during which latent frailty and cachexia-related vulnerability become clinically apparent.

From a clinical perspective, monitoring the ΔGNRI during the DTI may help identify patients at high risk of treatment intolerance before chemotherapy begins. The early recognition of accelerated nutritional decline could allow for the timely implementation of multidisciplinary supportive interventions, including nutritional support, the management of pancreatic exocrine insufficiency, biliary management, symptom control, and rehabilitation, with the aim of preserving treatment tolerability and treatment continuity. Malnutrition and metabolic disturbance are common in pancreatic cancer even before treatment initiation, and early nutritional assessment and intervention have already been emphasized as important aspects of care [27,28,29]. Our findings extend this concept by suggesting that, in addition to static nutritional assessment, a dynamic indicator such as the ΔGNRI may help identify patients with emerging cachexia-related vulnerability who are likely to benefit from early intervention.

This study has several limitations. First, it was a single-center retrospective study with a moderate sample size, and some estimates may have relatively wide confidence intervals. However, the consistency of the overall direction of the findings across multiple models and sensitivity analyses, including PS-matched analyses, supports the robustness of the main observations. Because this study was conducted at a single Japanese center with relatively prompt access to diagnostic imaging, biliary drainage, pathological confirmation, and chemotherapy initiation, the generalizability of our findings to other healthcare settings should be interpreted with caution. In healthcare systems with longer referral pathways or delayed access to supportive interventions, the DTI may be prolonged, potentially allowing for greater nutritional deterioration before chemotherapy initiation. Differences in referral pathways, nutritional support practices, chemotherapy selection, and patient characteristics across countries and institutions may influence the ΔGNRI trajectories and treatment outcomes; therefore, external validation in a multicenter cohort is warranted. Second, the exploratory the ΔGNRI cut-off value of −6.8 was derived from ROC curve analysis within the present single-center cohort. Therefore, this threshold may be subject to overfitting and should not be regarded as a definitive clinical decision threshold. Nevertheless, the main interpretation of this study was supported by analyses treating the ΔGNRI as a continuous variable, suggesting that the observed associations were not solely dependent on the selected cut-off value. Third, although propensity score matching was performed to minimize confounding, residual imbalance remained for distant metastasis, chemotherapy regimen, and total bilirubin. These imbalances may have incompletely accounted for confounding by disease burden and treatment selection, and the PS-matched findings should be interpreted as supportive rather than definitive. In addition, GnP and mFOLFIRINOX have different toxicity profiles, and regimen selection may have been influenced by patient fitness, performance status, organ function, and baseline nutritional condition. Although chemotherapy regimen was included in the multivariable models and propensity score model, residual confounding related to treatment selection and regimen-specific toxicity could not be fully excluded. Fourth, because of the retrospective design, we were unable to quantitatively disentangle the direct contributors to the GNRI decline during the DTI, such as invasive procedures, psychological stress, tumor-related catabolism, or other external stressors. In addition, cachexia was not assessed using formal diagnostic criteria, and longitudinal changes in skeletal muscle mass were not systematically evaluated. Therefore, the relationship between the ΔGNRI decline and cancer cachexia should be interpreted as hypothesis-generating. Fifth, the analysis was limited to patients who were able to initiate chemotherapy. As such, the impact of pre-treatment nutritional decline may have been underestimated because patients with the most severe deterioration may have become ineligible for systemic therapy before treatment initiation. Although PERT, opioid use, and dietary counseling during the DTI were assessed, detailed information on other nutritional and supportive interventions, including oral nutritional supplementation, quantitative dietary intake, and adherence to supportive interventions, was not systematically available. These factors may have influenced the nutritional trajectories, symptom burden, and treatment tolerability. Therefore, residual confounding related to supportive care could not be fully excluded. Finally, AEs were collected as a composite clinically significant endpoint, defined as grade ≥3 AEs or AEs requiring dose reduction, treatment interruption, hospitalization, or permanent discontinuation of first-line chemotherapy. Thus, we could not evaluate the overall burden of all-grade toxicities or perform grade-specific or symptom-specific analyses of gastrointestinal AEs. Future prospective multicenter studies with standardized nutritional, cachexia, and toxicity assessments are warranted.

## 5. Conclusions

Our findings indicate that dynamic nutritional decline during the DTI was associated with treatment intolerance, impaired treatment continuity, and poorer survival outcomes in patients with UR-PDAC. These findings suggest that the ΔGNRI may serve as a practical dynamic marker of pre-treatment nutritional and metabolic vulnerability beyond static nutritional status alone. Monitoring the ΔGNRI during this period may help identify patients who could benefit from early nutritional assessment, symptom management, and multidisciplinary supportive strategies before chemotherapy initiation.

## Figures and Tables

**Table 1:** Baseline characteristics of the overall cohort according to ΔGNRI status.

Characteristics		Δ Geriatric Nutritional Risk Index			
		Overall (*n* = 120)	≥−6.8 (*n* = 60)	<−6.8 (*n* = 60)	*p* Value
**Patient characteristics**					
Age, years		68.5 ± 7.4	68.6 ± 7.8	68.5 ± 7.1	0.955
Sex, *n* (%)					
	Female	46 (38)	24 (40)	22 (37)	0.851
	Male	74 (62)	36 (60)	38 (63)	
ECOG PS, *n* (%)					
	0	84 (70)	44 (73)	40 (67)	0.550
	1	36 (30)	16 (27)	20 (33)	
Body mass index, kg/m^2^		21.1 ± 2.8	21.3 ± 2.8	20.8 ± 2.9	0.302
Psoas muscle index, < −2SD, *n* (%)		29 (24)	12 (20)	17 (28)	0.394
**Tumor characteristics**					
Tumor location, *n* (%)					
	Head	64 (53)	26 (43)	38 (63)	0.043
	Body/tail	56 (47)	34 (57)	22 (37)	
Distant metastasis, present, *n* (%)		96 (80)	47 (78)	49 (82)	0.820
Ascites, present, *n* (%)		22 (18)	8 (13)	14 (23)	0.238
**Laboratory findings**					
Total bilirubin (mg/dL)		0.8 (0.6–1.5)	0.8 (0.6–2.0)	0.8 (0.6–1.5)	0.827
CA19-9 (U/mL)		541 (73–3228)	240 (65–1249)	959 (155–7392)	0.003
mGPS, *n* (%)					0.304
	0	68 (56)	37 (62)	31 (51)	
	1	38 (32)	15 (25)	23 (39)	
	2	14 (12)	8 (13)	6 (10)	
Initial-visit GNRI		97.2 ± 8.1	96.9 ± 8.2	97.6 ± 8.1	0.674
Pre-chemotherapy GNRI		90.2 ± 10.1	95.9 ± 8.2	84.5 ± 8.8	<0.001
ΔGNRI		−6.7 (−11.9–−1.1)	−1.1 (−4.5–0.9)	−11.9 (−15.8–−10.4)	<0.001
NLR		3.1 (2.2–5.0)	2.9 (2.1–5.0)	3.4 (2.6–4.9)	0.272
**Treatment characteristics**					
Biliary drainage during the DTI, *n* (%)		34 (28)	16 (27)	18 (30)	0.840
Chemotherapy regimen, *n* (%)					
	Gemcitabine + nab-paclitaxel	97 (81)	48 (80)	49 (82)	>0.999
	Modified FOLFIRINOX	23 (19)	12 (20)	11 (18)	
Diagnostic-to-treatment interval, months		0.8 (0.5–1.3)	0.8 (0.5–1.4)	0.8 (0.5–1.2)	0.733
**Supportive care during the DTI**					
PERT during the DTI, *n* (%)		13 (11)	7 (12)	6 (10)	1.000
Dietary counseling during the DTI, *n* (%)		18 (15)	11 (18)	7 (12)	0.444
Opioid use during the DTI, *n* (%)		26 (22)	9 (15)	17 (28)	0.120

Footnote: Values are presented as mean ± standard deviation, median (interquartile range), or number (%), as appropriate. ΔGNRI was defined as the GNRI immediately before the initiation of first-line chemotherapy minus the GNRI at the initial visit. The diagnostic-to-treatment interval was defined as the interval between the initial visit and initiation of first-line chemotherapy. *p* values were calculated using Student’s t-test or the Mann–Whitney U test for continuous variables and the χ^2^ test or Fisher’s exact test for categorical variables, as appropriate. A two-sided *p* value < 0.05 was considered statistically significant. Abbreviations: CA19-9, carbohydrate antigen 19-9; ECOG PS, Eastern Cooperative Oncology Group performance status; GNRI, Geriatric Nutritional Risk Index; ΔGNRI, change in Geriatric Nutritional Risk Index; NLR, neutrophil-to-lymphocyte ratio; DTI, diagnostic-to-treatment interval; mGPS, modified Glasgow Prognostic Score; SD, standard deviation; PERT, pancreatic enzyme replacement therapy.

**Table 2:** Treatment-related clinically significant adverse events, treatment discontinuation, and therapeutic sequencing according to ΔGNRI status in the entire cohort.

Variables	Δ Geriatric Nutritional Risk Index		
	≥−6.8 (*n* = 60)	<−6.8 (*n* = 60)	*p* Value
Early clinically significant AEs before first radiologic assessment			
Clinically significant non-hematologic AEs	15 (25)	32 (53)	0.002
Clinically significant gastrointestinal AEs	11 (18)	27 (45)	0.002
Clinically significant infectious AEs	6 (10)	9 (15)	0.582
Grade ≥3 hematologic AEs	15 (25)	6 (13)	0.163
Clinically significant AEs during the first-line therapy			
Clinically significant non-hematologic AEs	21 (35)	37 (62)	0.005
Clinically significant gastrointestinal AEs	13 (22)	30 (50)	0.021
Clinically significant infectious AEs	9 (15)	12 (20)	0.632
Grade ≥3 hematologic AEs	24 (40)	20 (33)	0.570
Hospitalization due to AEs	10 (17)	30 (50)	<0.001
Early treatment discontinuation before first radiologic assessment			
For any reason	4 (7)	21 (35)	<0.001
Due to AEs	0 (0)	13 (22)	<0.001
Treatment discontinuation during first-line therapy due to AEs	3 (5)	21 (35)	<0.001
Transition to second-line chemotherapy	44 (73)	29 (48)	0.008

Footnote: Values are presented as number (%). Clinically significant AEs were defined as grade ≥3 AEs or AEs requiring dose reduction, treatment interruption, hospitalization, or permanent discontinuation of first-line chemotherapy, with reference to CTCAE version 5.0. Early AEs were defined as clinically significant AEs occurring before the first radiologic response evaluation. AEs during first-line therapy were defined as clinically significant AEs occurring at any time from the initiation to discontinuation of first-line chemotherapy. Hematologic AEs were tabulated as grade ≥3 events according to CTCAE version 5.0. Non-hematologic AEs included all clinically significant AEs other than hematologic AEs. Gastrointestinal AEs were defined as clinically significant AEs categorized under “Gastrointestinal disorders” in CTCAE and were analyzed as a grouped category rather than as individual symptoms. Infectious AEs included clinically significant infections, including cholangitis. Hospitalization due to AEs included admissions for management of treatment-related AEs. Comparisons between groups were performed using the χ^2^ test or Fisher’s exact test, as appropriate. A two-sided *p* value < 0.05 was considered statistically significant. Abbreviations: AEs, adverse events; CTCAE, Common Terminology Criteria for Adverse Events; ΔGNRI, change in Geriatric Nutritional Risk Index.

**Table 3:** Multivariable logistic regression analyses for early treatment discontinuation and failure to transition to second-line therapy.

Outcome	Variable	OR (95% CI)	*p* Value
Early Treatment Discontinuation	ΔGNRI (per 1-point increase)	0.89 (0.83–0.96)	<0.001
	ECOG PS (1 vs. 0)	3.48 (1.31–9.22)	0.012
Failure to Transition to Second-line Chemotherapy	ΔGNRI (per 1-point increase)	0.92 (0.87–0.98)	0.009
	ECOG PS (1 vs. 0)	1.75 (0.73–4.16)	0.209
	Ascites (Present vs. Absent)	3.26 (1.16–9.17)	0.025
	Regimen (mFOLFIRINOX vs. GnP)	0.49 (0.17–1.49)	0.213

Footnotes: Odds ratios (ORs) are presented per 1-point increase in ΔGNRI (i.e., a higher ΔGNRI indicates less nutritional deterioration). Early treatment discontinuation was defined as the cessation of first-line chemotherapy before the first radiologic response evaluation. Failure to transition to second-line therapy was defined as inability to initiate any second-line chemotherapy after discontinuation of the first-line therapy. ECOG performance status was dichotomized as 1 versus 0. Regimen was categorized as gemcitabine plus nab-paclitaxel (GnP) versus modified FOLFIRINOX (mFOLFIRINOX). Odds ratios are shown with 95% confidence intervals. A two-sided *p* value <0.05 was considered statistically significant. Abbreviations: CI, confidence interval; ECOG PS, Eastern Cooperative Oncology Group performance status; GnP, gemcitabine plus nab-paclitaxel; mFOLFIRINOX, modified FOLFIRINOX; ΔGNRI, change in Geriatric Nutritional Risk Index; OR, odds ratio.

**Table 4:** Multivariable Cox proportional hazards analyses for time to treatment failure and overall survival.

Outcome	Variable	HR (95% CI)	*p* Value
Overall Survival	ΔGNRI (per 1-point increase)	0.96 (0.93–0.98)	0.004
	ECOG PS (1 vs. 0)	1.62 (1.06–2.48)	0.024
	Regimen (mFOLFIRINOX vs. GnP)	0.96 (0.58–1.57)	0.877
Time to Treatment Failure	ΔGNRI (per 1-point increase)	0.96 (0.94–0.99)	0.009
	ECOG PS (1 vs. 0)	1.46 (0.96–2.21)	0.074
	Regimen (mFOLFIRINOX vs. GnP)	1.25 (0.77–2.02)	0.360

Footnotes: Time to treatment failure was defined as the interval from the initiation of first-line chemotherapy to treatment discontinuation, disease progression, or death from any cause, whichever occurred first. Overall survival was defined as the interval from the initiation of first-line chemotherapy to death from any cause. ΔGNRI represents the change in GNRI from the initial visit to immediately before the initiation of first-line chemotherapy; negative values indicate nutritional deterioration. ECOG performance status was restricted to 0–1, as patients with ECOG PS ≥2 were not eligible for combination chemotherapy at our institution. Hazard ratios are shown with 95% confidence intervals. A two-sided *p* value <0.05 was considered statistically significant. Abbreviations: CI, confidence interval; ECOG PS, Eastern Cooperative Oncology Group performance status; GnP, gemcitabine plus nab-paclitaxel; mFOLFIRINOX, modified FOLFIRINOX; GNRI, Geriatric Nutritional Risk Index; ΔGNRI, change in Geriatric Nutritional Risk Index; HR, hazard ratio.

## Data Availability

The datasets generated and/or analyzed during the current study are not publicly available because they contain potentially identifiable clinical information but are available from the corresponding author on reasonable request and subject to institutional approval.

## Supplementary Materials

The following supporting information can be downloaded at https://www.mdpi.com/article/10.3390/nu18121998/s1. Figure S1: Distribution of ΔGNRI and determination of the exploratory cut-off value. Figure S2: Longitudinal changes in GNRI according to ΔGNRI status. Figure S3: Kaplan–Meier analyses for time to treatment failure and overall survival in the propensity score-matched cohort stratified by ΔGNRI status. Figure S4: Relationship between dynamic changes in GNRI and NLR during the diagnostic-to-treatment interval. Table S1: Full baseline characteristics of the overall cohort according to ΔGNRI status. Table S2: Baseline characteristics of the analyzed cohort and excluded patients. Table S3: Baseline characteristics of the propensity score-matched cohort according to ΔGNRI status. Table S4: Longitudinal changes in nutritional indices during the diagnostic-to-treatment interval and early treatment period. Table S5: Primary endpoints in the propensity score-matched cohort according to ΔGNRI status. Table S6: Sensitivity multivariable logistic regression models for early treatment discontinuation across sequentially adjusted models. Table S7: Sensitivity multivariable analyses for failure to transition to second-line chemotherapy. Table S8A: Adjusted Cox proportional hazards models for survival outcomes. Table S8B: Full multivariable Cox regression models for overall survival and time to treatment failure. Table S9: Exploratory Cox interaction analyses evaluating whether DTI duration modified the association between ΔGNRI and survival outcomes. Table S10: Comparison of dynamic (ΔGNRI) and static pre-chemotherapy GNRI in multivariable models for early treatment discontinuation. Table S11: Sensitivity analyses incorporating ΔGNRI, ΔNLR, and total bilirubin for early treatment discontinuation.

## Abbreviations

The following abbreviations are used in this manuscript:

CA19-9	Carbohydrate antigen 19-9
CI	Confidence interval
SMD	Standardized mean difference
PNI	Prognostic Nutritional Index
IQR	Interquartile range
SD	Standard deviation
CT	Computed tomography
ANOVA	Analysis of variance
PERT	Pancreatic enzyme replacement therapy
UR-PDAC	Unresectable pancreatic ductal adenocarcinoma
PDAC	Pancreatic ductal adenocarcinoma
DTI	Diagnostic-to-treatment interval
GNRI	Geriatric Nutritional Risk Index
TTF	Time to treatment failure
OS	Overall survival
GnP	Gemcitabine plus nab-paclitaxel
AEs	Adverse events
mFOLFIRINOX	modified FOLFIRINOX
ECOG PS	Eastern Cooperative Oncology Group performance status
NLR	Neutrophil-to-lymphocyte ratio
mGPS	modified Glasgow Prognostic Score
PMI	Psoas muscle index
CTCAE	Common Terminology Criteria for Adverse Events
