# Supplementary material for: Dynamic Nutritional Decline During the Diagnostic-to-Treatment Interval Is Associated with Treatment Resilience and Survival in Unresectable Pancreatic Ductal Adenocarcinoma
Source: Nutrients. 2026 Jun 19;18(12):1998. doi: 10.3390/nu18121998 (PMC13305834; doi:10.3390/nu18121998)
Supplement: Supplementary file 1 [file nutrients-18-01998-s001.zip › nutrients-4378554-supplementary.pdf]

Supplementary Materials

Figure Legends

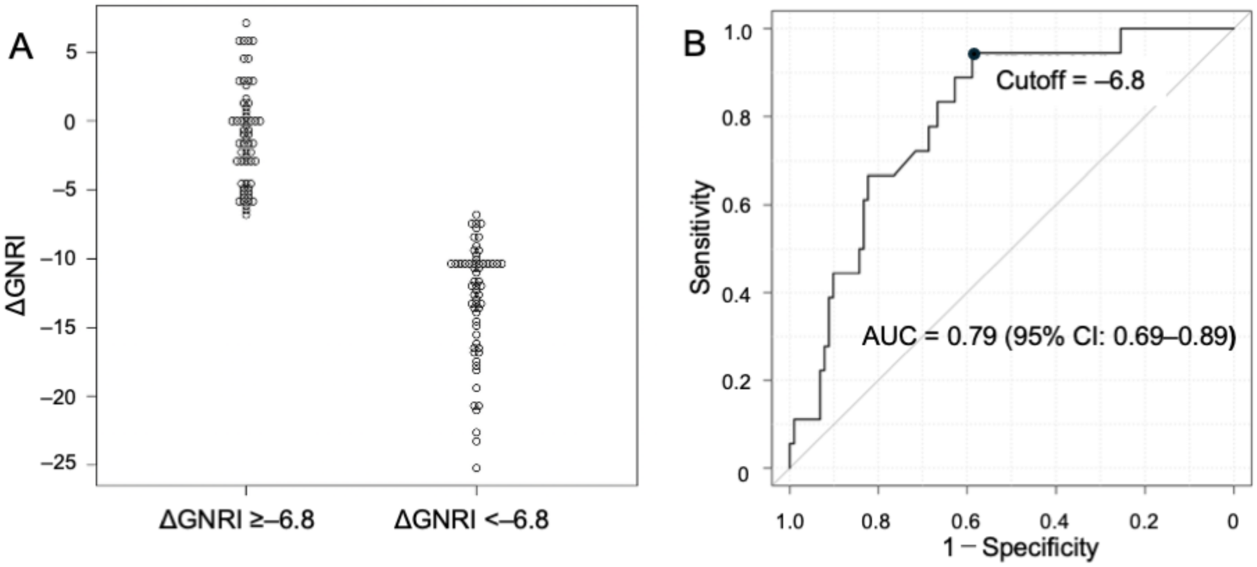

Supplementary Figure S1. Distribution of ΔGNRI and determination of the exploratory cut-off value.

(A) Distribution of ΔGNRI during the diagnostic-to-treatment interval, shown separately for the GNRI-maintained (ΔGNRI ≥ −6.8) and GNRI-decreased (ΔGNRI < −6.8) groups.

(B) Receiver operating characteristic curve analysis of ΔGNRI for predicting early treatment discontinuation. The exploratory cut-off value of −6.8 was determined using the Youden index (sensitivity, 57.8%; specificity, 94.4%; area under the curve, 0.79; 95% confidence interval, 0.69–0.89). Abbreviations: DTI, diagnostic-to-treatment interval; GNRI, Geriatric Nutritional Risk Index; ΔGNRI, change in Geriatric Nutritional Risk Index.

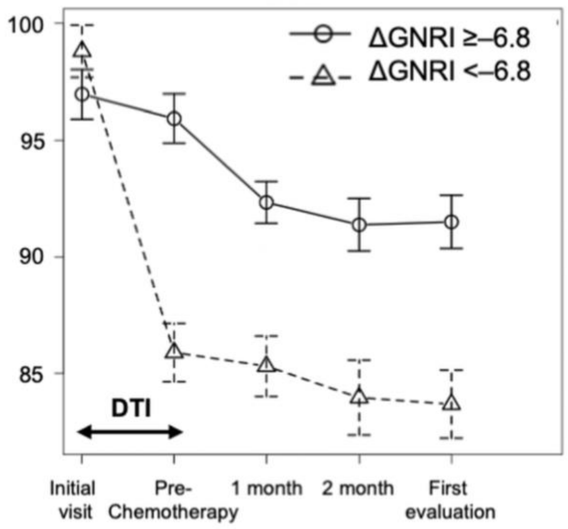

Supplementary Figure S2. Longitudinal changes in GNRI according to ΔGNRI status.

Mean GNRI values over time are shown for patients in the GNRI-maintained group (ΔGNRI ≥ −6.8) and the GNRI-decreased group (ΔGNRI < −6.8). Measurements were obtained at the initial visit, immediately before initiation of first-line chemotherapy (pre-chemotherapy), and at 1 month, 2 months, and the first radiologic evaluation after treatment initiation. Error bars represent standard errors. A significant group × time interaction was observed in a two-way repeated-measures analysis of variance (Greenhouse–Geisser corrected $p < 0.001$). Abbreviations: GNRI, Geriatric Nutritional Risk Index; ΔGNRI, change in Geriatric Nutritional Risk Index; DTI, diagnostic-to-treatment interval.

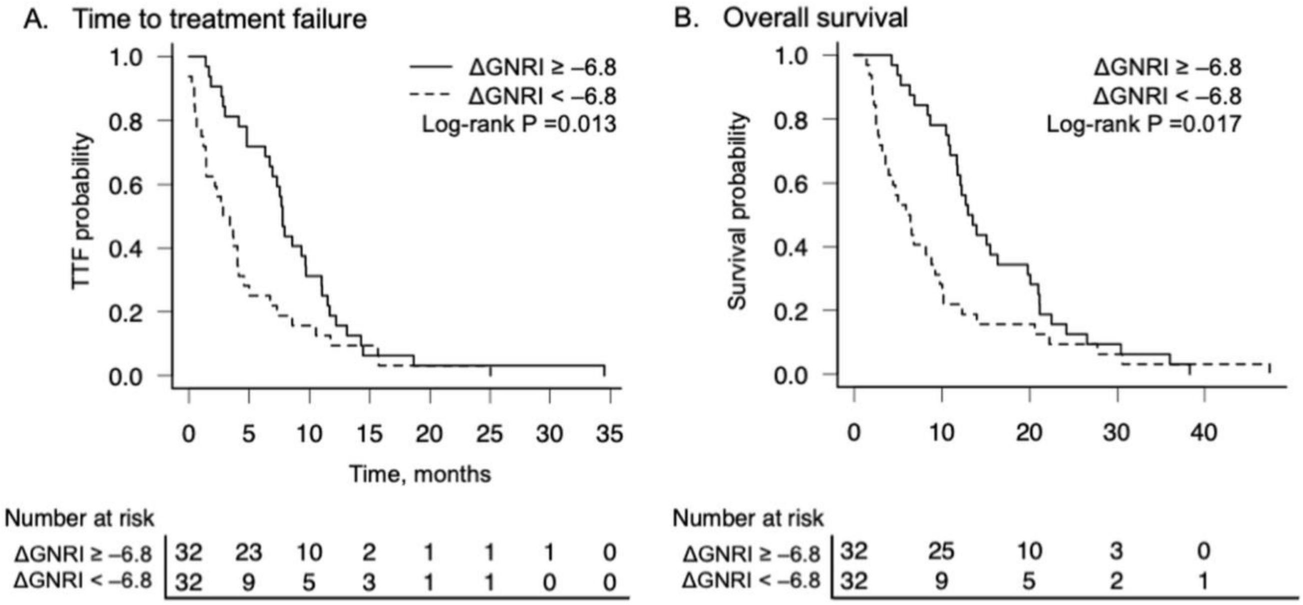

Supplementary Figure S3. Kaplan–Meier analyses for time to treatment failure and overall survival in the propensity score–matched cohort stratified by ΔGNRI status.

Kaplan–Meier curves comparing (A) time to treatment failure (TTF) and (B) overall survival (OS) between patients with ΔGNRI ≥ −6.8 and those with ΔGNRI < −6.8 in the propensity score–matched cohort. Median survival times were as follows: TTF, 7.8 months (95% CI, 6.3–9.7) in the ΔGNRI ≥ −6.8 group and 3.1 months (95% CI, 1.4–4.1) in the ΔGNRI < −6.8 group; OS, 13.2 months (95% CI, 10.9–19.7) in the ΔGNRI ≥ −6.8 group and 6.2 months (95% CI, 3.2–9.2) in the ΔGNRI < −6.8 group. Survival distributions were compared using the log-rank test. Numbers at risk are shown beneath each plot. Abbreviations: CI, confidence interval; GNRI, Geriatric Nutritional Risk Index; ΔGNRI, change in Geriatric Nutritional Risk Index; OS, overall survival; TTF, time to treatment failure.

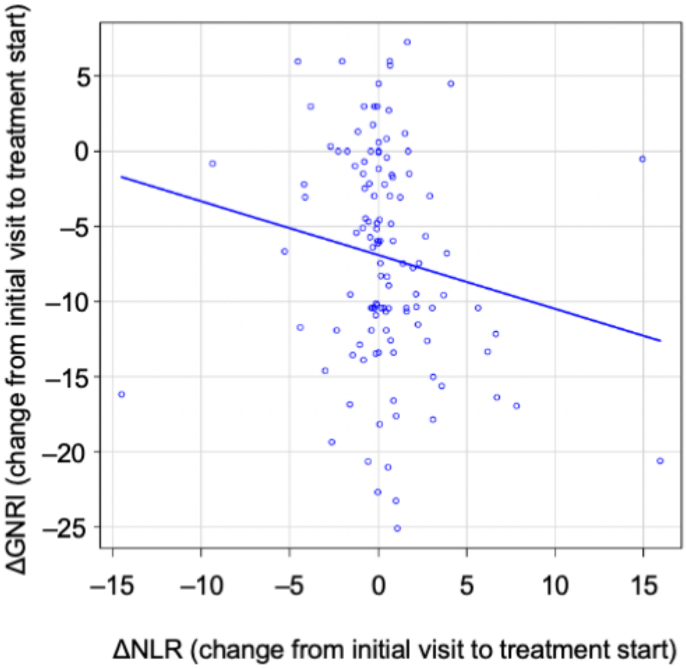

Supplementary Figure S4. Relationship between dynamic changes in GNRI and NLR during the diagnostic-to-treatment interval.

A scatter plot showing the relationship between changes in the Geriatric Nutritional Risk Index (ΔGNRI) and changes in the neutrophil-to-lymphocyte ratio (ΔNLR) from the initial visit to initiation of first-line chemotherapy. The solid line represents the fitted linear regression line. Pearson's correlation coefficient was r = −0.32 (95% CI, −0.49 to −0.13; *p* = 0.001). Abbreviations: CI, confidence interval; GNRI, Geriatric Nutritional Risk Index; ΔGNRI, change in Geriatric Nutritional Risk Index; NLR, neutrophil-to-lymphocyte ratio; ΔNLR, change in neutrophil-to-lymphocyte ratio.

Supplementary Table S1. Full baseline characteristics of the overall cohort according to ΔGNRI status.

Characteristics	Overall (n=120)	Δ Geriatric Nutritional Risk Index ≥ −6.8 (*n* =60)	< −6.8 (*n* = 60)	*p* Value
**Patient characteristics**				
Age, years	68.5 ± 7.4	68.6 ± 7.8	68.5 ± 7.1	0.955
Sex, *n* (%)				
Female	46 (38)	24 (40)	22 (37)	0.851
Male	74 (62)	36 (60)	38 (63)	
ECOG PS, *n* (%)				
0	84 (70)	44 (73)	40 (67)	0.550
1	36 (30)	16 (27)	20 (33)	
Weight, kg	54.5 ± 9.4	54.7 ± 9.6	54.2 ± 9.2	0.771
Body mass index, kg/m^2	21.1 ± 2.8	21.3 ± 2.8	20.8 ± 2.9	0.302
Psoas muscle index, < −2SD, *n* (%)	29 (24)	12 (20)	17 (28)	0.394
**Tumor characteristics**				
Tumor location, *n* (%)				
Head	64 (53)	26 (43)	38 (63)	0.043
Body/tail	56 (47)	34 (57)	22 (37)	
Stage, *n* (%)				
IIA/IIB	4 (3)	2 (3)	2 (3)	0.787
III	20 (17)	11 (18)	9 (15)	
IV	96 (80)	47 (79)	49 (81)	
Distant metastasis, present, *n* (%)	96 (80)	47 (78)	49 (82)	0.820
Metastatic site, *n* (%)				
Liver	54 (45)	22 (36)	32 (54)	0.098
Lung	27 (23)	12 (20)	15 (25)	0.662
Peritoneal seeding	24 (20)	13 (21)	11 (19)	0.820
Multiple organs	21 (18)	7 (12)	14 (24)	0.148
Jaundice, present, *n* (%)	34 (28)	16 (26)	18 (31)	0.840
Ascites, present, *n* (%)	22 (18)	8 (13)	14 (23)	0.238
**Laboratory findings**				
Albumin (g/dL)	4.0 (3.7–4.2)	4.0 (3.7–4.2)	4.0 (3.7–4.3)	0.248
Total bilirubin (mg/dL)	0.8 (0.6–1.5)	0.8 (0.6–2.0)	0.8 (0.6–1.5)	0.827
Creatinine (mg/dL)	0.7 (0.6–0.8)	0.7 (0.6–0.8)	0.7 (0.6–0.8)	0.085
C-reactive protein (mg/dL)	0.3 (0.1–1.3)	0.2 (0.1–0.9)	0.5 (0.2–1.7)	0.176
HbA1c (%)	6.2 (5.8–7.5)	6.4 (5.8–7.8)	6.2 (5.8–7.4)	0.532
White blood cell count (/µL)	6550 (5375–8825)	6400 (5150–7750)	6600 (5475–8525)	0.429
Hemoglobin (g/dL)	13.2 (11.7–14.0)	12.9 (11.4–14.0)	13.3 (12.1–14.1)	0.186
Platelet count (×10^3/µL)	224 (184–279)	221 (181–275)	228 (203–290)	0.248
CEA (ng/mL)	4.6 (2.7–13.2)	4.4 (2.6–9.9)	5.5 (2.8–17.9)	0.343
CA19-9 (U/mL)	541 (73–3228)	240 (65–1249)	959 (155–7392)	0.003
mGPS, *n* (%)				0.304
0	68 (56)	37 (62)	31 (51)	
1	38 (32)	15 (25)	23 (39)	
2	14 (12)	8 (13)	6 (10)	
PNI	46.7 ± 6.4	46.3 ± 6.7	47.1 ± 6.2	0.526
Initial-visit GNRI	97.2 ± 8.1	96.9 ± 8.2	97.6 ± 8.1	0.674
Pre-chemotherapy GNRI	90.2 ± 10.1	95.9 ± 8.2	84.5 ± 8.8	<0.001
ΔGNRI	−6.7 (−11.9–−1.1)	−1.1 (−4.5–0.9)	−11.9 (−15.8–−10.4)	<0.001
NLR	3.1 (2.2–5.0)	2.9 (2.1–5.0)	3.4 (2.6–4.9)	0.272
**Treatment characteristics**				
Biliary drainage during the DTI, *n* (%)	34 (28)	16(27)	18 (30)	0.840
Chemotherapy regimen, *n* (%)				
Gemcitabine+nab-paclitaxel	97 (81)	48 (80)	49 (82)	>0.999
Modified FOLFIRINOX	23 (19)	12 (20)	11 (18)	
Diagnostic-to-treatment interval, months	0.8 (0.5–1.3)	0.8 (0.5–1.4)	0.8 (0.5–1.2)	0.733
**Supportive care during the DTI**				
PERT during the DTI, *n* (%)	13 (11)	7 (12)	6 (10)	1.000
Dietary counseling during the DTI, *n* (%)	18 (15)	11 (18)	7 (12)	0.444
Opioid use during the DTI, *n* (%)	26 (22)	9 (15)	17 (28)	0.120

Footnotes: Continuous variables are presented as mean ± standard deviation or median (interquartile range), and categorical variables as number (%), as appropriate. ΔGNRI represents the change in GNRI from the initial visit to immediately before initiation of first-line chemotherapy. Abbreviations: CA19-9, carbohydrate antigen 19-9; ECOG PS, Eastern Cooperative Oncology Group performance status; GNRI, Geriatric Nutritional Risk Index; ΔGNRI, change in Geriatric Nutritional Risk Index; NLR, neutrophil-to-lymphocyte ratio; IQR, interquartile range; mGPS, modified Glasgow Prognostic Score; PNI, Prognostic Nutritional Index; SD, standard deviation; PERT, pancreatic enzyme replacement therapy.

Supplementary Table S2. Baseline characteristics of the analyzed cohort and excluded patients.

Characteristics	Analyzed cohort (n = 120)	Excluded patients (n = 12)
**Patient characteristics**		
Age, years	68.5 ± 7.4	67.3 ± 9.0
Sex, n (%)		
Female	46 (38)	7 (58)
Male	74 (62)	5 (42)
ECOG PS, n (%)		
0	84 (70)	8 (67)
1	36 (30)	4 (33)
Weight, kg	54.5 ± 9.4	55.4 ± 12.8
Body mass index, kg/m^2	21.1 ± 2.8	21.9 ± 3.1
Psoas muscle index, < −2SD, n (%)	29 (24)	4 (33)
**Tumor characteristics**		
Tumor location, n (%)		
Head	64 (53)	2 (17)
Body/tail	56 (47)	10 (83)
Stage, n (%)		
IIA/IIB	4 (3)	1 (8)
III	20 (17)	4 (33)
IV	96 (80)	7 (58)
Distant metastasis, present, n (%)	96 (80)	7 (58)
Metastatic site, n (%)		
Liver	54 (45)	7 (58)
Lung	27 (23)	2 (17)
Peritoneal seeding	24 (20)	4 (33)
Multiple organs	21 (18)	4 (33)
Jaundice, present, n (%)	34 (28)	1 (8)
Ascites, present, n (%)	22 (18)	2 (17)
**Laboratory findings**		
Albumin (g/dL)	4.0 (3.7–4.2)	3.8 (3.7–4.0)
Total bilirubin (mg/dL)	0.8 (0.6–1.5)	1.0 (0.5–1.1)
C-reactive protein (mg/dL)	0.3 (0.1–1.3)	0.3 (0.05–0.76)
HbA1c (%)	6.2 (5.8–7.5)	5.8 (5.6–6.7)
White blood cell count (/μL)	6550 (5375–8825)	6850 (5325–8325)
Hemoglobin (g/dL)	13.2 (11.7–14.0)	12.5 (11.3–14.1)
Platelet count (×10^3/μL)	224 (184–279)	228 (195–264)
CEA (ng/mL)	4.6 (2.7–13.2)	8.2 (2.7–30.0)
CA19-9 (U/mL)	541 (73–3228)	270 (14–3814)
mGPS, n (%)		
0	68 (56)	8 (67)
1	38 (32)	3 (25)
2	14 (12)	1 (8)
PNI	46.7 ± 6.4	45.1 ± 5.1
Initial-visit GNRI	97.2 ± 8.1	96.2 ± 6.1
Pre-chemotherapy GNRI	90.2 ± 10.1	90.5 ± 8.6
ΔGNRI	−6.7 (−11.9–−1.1)	−7.4 (−16.4–−3.6)
NLR	3.1 (2.2–5.0)	4.0 (2.7–4.5)
**Treatment characteristics**		
Biliary drainage during the DTI, n (%)	34 (28)	1 (8)
Reason for exclusion		
Discontinued chemotherapy at patient preference for reasons unrelated to adverse events		4 (33)
Received concomitant radiotherapy		5 (42)
Continued treatment at other institutions		3 (25)

Footnotes: Values are presented as mean ± standard deviation, median (interquartile range), or number (%), as appropriate. Metastatic sites were not mutually exclusive. Psoas muscle index < −2SD was defined as <6.36 cm²/m² in men and <3.92 cm²/m² in women. Abbreviations: CA19-9, carbohydrate antigen 19-9; CEA, carcinoembryonic antigen;

DTI, diagnostic-to-treatment interval; ECOG PS, Eastern Cooperative Oncology Group performance status; GNRI, Geriatric Nutritional Risk Index; ΔGNRI, change in Geriatric Nutritional Risk Index; NLR, neutrophil-to-lymphocyte ratio; HbA1c, glycated hemoglobin; mGPS, modified Glasgow Prognostic Score; PNI, Prognostic Nutritional Index.

Supplementary Table S3. Baseline characteristics of the propensity score–matched cohort according to ΔGNRI status.

Characteristics	Overall (*n* = 64)	Δ Geriatric Nutritional Risk Index ≥ −6.8 (*n* = 32)	Δ Geriatric Nutritional Risk Index < −6.8 (*n* = 32)	Standardized mean difference
Age, years	68.2 ± 7.7	67.5 ± 7.8	68.9 ± 7.6	0.196
Sex, *n* (%)				
Female	23 (36)	12 (37)	11 (34)	0.063
Male	41 (64)	20 (63)	21 (66)	
ECOG PS, *n* (%)				
0	38 (59)	20 (63)	18 (56)	0.128
1	26 (41)	12 (37)	14 (44)	
Psoas muscle index, < −2SD, *n* (%)	19 (30)	8 (25)	11 (31)	0.139
Tumor location, *n* (%)				
Head	33 (52)	16 (50)	17 (53)	0.063
Body/tail	31 (48)	16 (50)	15 (47)	
Distant metastasis, present, *n* (%)	53 (82)	25 (78)	28 (87)	0.250
Ascites, present, *n* (%)	12 (19)	5 (16)	7 (21)	0.161
Total bilirubin (mg/dL)	0.8 (0.6–1.5)	0.9 (0.6–3.5)	0.8 (0.6–0.9)	0.440
CA19-9 (U/mL)	903 (110–4028)	988 (107–2161)	890 (134–7392)	0.032
mGPS, *n* (%)				0.140
0	36 (56)	19 (59)	17 (53)	
1	20 (32)	9 (28)	11 (34)	
2	8 (12)	4 (13)	4 (13)	
Biliary drainage during the DTI, *n* (%)	19 (30)	12 (38)	7 (22)	0.347
Chemotherapy regimen, *n* (%)				
Gemcitabine+nab-paclitaxel	53 (83)	25 (78)	28 (87)	0.250
Modified FOLFIRINOX	11 (17)	7 (22)	4 (13)	
Diagnostic-to-treatment interval, months	0.8 (0.5–1.3)	0.8 (0.5–1.2)	0.8 (0.5–1.3)	0.158

Footnotes: Propensity scores were estimated using a logistic regression model incorporating clinically relevant covariates, as described in the Methods. ΔGNRI was defined as the GNRI immediately before initiation of first-line chemotherapy minus the GNRI at the initial visit. Continuous variables are presented as mean ± standard deviation or median (interquartile range), as appropriate; categorical variables are presented as number (%). Standardized mean differences (SMDs) <0.20 were considered indicative of adequate covariate balance. Although most covariates achieved adequate balance (SMD <0.20), residual imbalance was observed for distant metastasis, biliary drainage during the DTI, chemotherapy regimen, and total bilirubin (SMD >0.20), which should be considered when interpreting results from the propensity score-matched analyses. Abbreviations: **S**MD, standardized mean difference; ECOG PS, Eastern Cooperative Oncology Group performance status; CA19-9, carbohydrate antigen 19-9; mGPS, modified Glasgow Prognostic Score; GNRI, Geriatric Nutritional Risk Index; ΔGNRI, change in Geriatric Nutritional Risk Index; NLR, neutrophil-to-lymphocyte ratio; DTI, diagnostic-to-treatment interval; IQR, interquartile range; SD, standard deviation.

Supplementary Table S4. Longitudinal changes in nutritional indices during the diagnostic-to-treatment interval and early treatment period.

Timepoint	Δ Geriatric Nutritional Risk Index ≥ −6.8 (*n* = 60)	< −6.8 (*n* = 60)	*p* (interaction)
GNRI			**<0.001**
Initial visit	96.9 ± 8.2	97.6 ± 8.1	
Pre-chemotherapy	95.9 ± 8.2	84.5 ± 8.8	
1 month	92.3 ± 6.9	83.1 ± 9.4	
2 months	91.4 ± 8.7	82.4 ± 11.9	
First evaluation	91.5 ± 8.8	83.2 ± 10.1	
Body weight (kg)			**0.028**
Initial visit	54.7 ± 9.7	54.2 ± 9.2	
Pre-chemotherapy	53.9 ± 9.3	52.3 ± 9.0	
1 month	53.7 ± 9.3	50.7 ± 8.9	
2 months	53.1 ± 9.9	52.1 ± 9.4	
First evaluation	53.1 ± 9.6	51.3 ± 9.7	
Serum albumin (g/dL)			**<0.001**
Initial visit	4.0 (3.7–4.2)	4.0 (3.8–4.3)	
Pre-chemotherapy	3.9 (3.5–4.1)	3.3 (3.0–3.5)	
1 month	3.6 (3.3–3.8)	3.2 (2.8–3.5)	
2 months	3.6 (3.4–3.8)	3.3 (2.8–3.5)	
First evaluation	3.6 (3.3–3.9)	3.3 (2.8–3.5)	

Body weight and serum albumin were measured on the same day at each time point. GNRI was calculated at each time point using concurrently obtained body weight and serum albumin.

Normally distributed variables are presented as mean ± standard deviation, and skewed variables as median (interquartile range). First evaluation refers to the first radiologic response assessment after chemotherapy initiation.

p values represent group × time interaction effects derived from two-way repeated-measures analysis of variance with Greenhouse–Geisser correction for violation of sphericity. Abbreviations: GNRI, Geriatric Nutritional Risk Index; IQR, interquartile range; SD, standard deviation; DTI, diagnostic-to-treatment interval.

Supplementary Table S5. Primary endpoints in the propensity score-matched cohort according to ΔGNRI status.

Outcome	Δ Geriatric Nutritional Risk Index ≥ −6.8 (*n* = 32)	< −6.8 (*n* = 32)	p Value
Early treatment discontinuation, n (%)	3 (9.4)	14 (43.8)	0.004
Failure to transition to second-line chemotherapy, n (%)	9 (28.1)	24 (75.0)	<0.001

Values are presented as number (%). Patients were matched 1:1 using nearest-neighbor propensity score matching without replacement. Early treatment discontinuation was defined as cessation of first-line chemotherapy before the first radiologic response evaluation. Failure to transition to second-line therapy was defined as inability to initiate any second-line chemotherapy after discontinuation of first-line therapy. Group comparisons were performed using Fisher's exact test. A two-sided p value <0.05 was considered statistically significant. Abbreviations: GNRI, Geriatric Nutritional Risk Index; ΔGNRI, change in Geriatric Nutritional Risk Index.

Supplementary Table S6. Sensitivity multivariable logistic regression models for early treatment discontinuation across sequentially adjusted models.

Model	Additional Covariates	OR (95% CI)	p Value
Base	ECOG PS	0.89 (0.83–0.96)	<0.001
A	+ Ascites	0.89 (0.82–0.95)	0.002
B	+ Distant metastasis	0.89 (0.83–0.96)	0.001
C	+ Total bilirubin	0.88 (0.82–0.96)	<0.001
D	+ Ascites + Total bilirubin	0.88 (0.82–0.95)	0.001
E	+ Ascites + Distant metastasis	0.89 (0.83–0.96)	0.002

Odds ratios (ORs) are presented per 1-point increase in ΔGNRI. The base model included ECOG performance status. Subsequent models incorporated additional clinically relevant covariates, including ascites, distant metastasis, and total bilirubin. Early treatment discontinuation was defined as cessation of first-line chemotherapy before the first radiologic response evaluation. Odds ratios are shown with 95% confidence intervals. A two-sided *p* value <0.05 was considered statistically significant. Abbreviations: CI, confidence interval; ECOG PS, Eastern Cooperative Oncology Group performance status; GNRI, Geriatric Nutritional Risk Index; ΔGNRI, change in Geriatric Nutritional Risk Index; OR, odds ratio.

Supplementary Table S7. Sensitivity multivariable analyses for failure to transition to second-line chemotherapy.

Model	Additional Covariates	OR (95% CI)	p Value
Base	ECOG PS, Ascites, Regimen	0.92 (0.87–0.98)	0.009
A	+ Total bilirubin	0.93 (0.88–0.98)	0.009
B	+ Distant metastasis	0.93 (0.88–0.98)	0.010
C	ΔGNRI < −6.8 (vs ≥ −6.8)	2.72 (1.23–6.02)	0.013

The base model included ECOG performance status, ascites, and chemotherapy regimen. Additional models incorporated total bilirubin and distant metastasis. ΔGNRI was analyzed both as a continuous variable (per 1-point increase) and as a categorical variable using the exploratory cut-off value of −6.8. Failure to transition to second-line therapy was defined as inability to initiate any second-line chemotherapy after discontinuation of first-line therapy. Odds ratios are presented with 95% confidence intervals. A two-sided *p* value <0.05 was considered statistically significant. Abbreviations: CI, confidence interval; ECOG PS, Eastern Cooperative Oncology Group performance status; GNRI, Geriatric Nutritional Risk Index; ΔGNRI, change in Geriatric Nutritional Risk Index; OR, odds ratio.

Supplementary Table S8A. Adjusted Cox proportional hazards models for survival outcomes.

Adjusted Cox proportional hazards models evaluating the association between ΔGNRI and survival outcomes across progressively adjusted models.

Outcome	Model	Additional Covariates	HR (95% CI)	p Value
Overall Survival	Base	ECOG PS + Regimen	0.95 (0.93–0.98)	0.003
	A	+ Ascites	0.95 (0.93–0.98)	0.003
	B	+ Distant metastasis	0.95 (0.93–0.98)	<0.001
	C	+ Total bilirubin	0.96 (0.93–0.98)	0.004
	D	+ Regimen + ΔGNRI×Regimen interaction	0.95 (0.92–0.98)	0.002
Time to Treatment Failure	Base	ECOG PS + Regimen	0.96 (0.94–0.99)	0.009
	A	+ Ascites	0.96 (0.94–0.99)	0.008
	B	+ Distant metastasis	0.96 (0.94–0.99)	0.008
	C	+ Total bilirubin	0.96 (0.94–0.99)	0.016
	D	+ Regimen + ΔGNRI×Regimen interaction	0.96 (0.94–0.99)	0.012

Footnotes:

Hazard ratios (HRs) are presented per 1-point increase in ΔGNRI. The base model included ECOG performance status and chemotherapy regimen. Subsequent models additionally adjusted for ascites, distant metastasis, total bilirubin, and the interaction term between ΔGNRI and chemotherapy regimen. No statistically significant interaction between ΔGNRI and chemotherapy regimen was observed. Hazard ratios are presented with 95% confidence intervals. A two-sided *p* value <0.05 was considered statistically significant. Abbreviations: CI, confidence interval; ECOG PS, Eastern Cooperative Oncology Group performance status; GNRI, Geriatric Nutritional Risk Index; ΔGNRI, change in Geriatric Nutritional Risk Index; HR, hazard ratio; OS, overall survival; TTF, time to treatment failure.

Supplementary Table S8B. Full multivariable Cox regression models for overall survival and time to treatment failure.

Full multivariable Cox proportional hazards regression models for OS and TTF.

Outcome	Model	Variable	HR (95% CI)	*p* Value
Overall Survival	Base			
		ΔGNRI (per 1-point increase)	0.96 (0.93–0.98)	0.004
		ECOG PS (1 vs 0)	1.62 (1.06–2.48)	0.024
		Regimen (mFOLFIRINOX vs GnP)	0.96 (0.58–1.57)	0.877
	Model A			
		ΔGNRI (per 1-point increase)	0.95 (0.93–0.98)	0.003
		ECOG PS (1 vs 0)	1.70 (1.12–2.59)	0.013
		Regimen (mFOLFIRINOX vs GnP)	0.96 (0.59–1.57)	0.900
		Ascites (Present vs Absent)	2.70 (1.67–4.37)	<0.001
	Model B			
		ΔGNRI (per 1-point increase)	0.95 (0.93–0.98)	<0.001
		ECOG PS (1 vs 0)	1.49 (0.98–2.29)	0.061
		Regimen (mFOLFIRINOX vs GnP)	1.07 (0.66–1.75)	0.776
		Distant metastasis (Present vs Absent)	2.94 (1.74–4.97)	<0.001
	Model C			
		ΔGNRI (per 1-point increase)	0.96 (0.93–0.98)	0.004
		ECOG PS (1 vs 0)	1.60 (1.05–2.45)	0.029
		Regimen (mFOLFIRINOX vs GnP)	0.95 (0.58–1.56)	0.851
		Total bilirubin (per 1mg/dL increase)	0.98 (0.96–1.02)	0.536
	Model D			
		ΔGNRI (per 1-point increase)	0.95 (0.92–0.98)	0.002
		Regimen (mFOLFIRINOX vs GnP)	1.36 (0.67–2.74)	0.386
		ΔGNRI×Regimen interaction	1.06 (0.98–1.13)	0.102
Time to Treatment Failure	Base			
		ΔGNRI (per 1-point increase)	0.96 (0.94–0.99)	0.009
		ECOG PS (1 vs 0)	1.46 (0.96–2.21)	0.074
		Regimen (mFOLFIRINOX vs GnP)	1.25 (0.77–2.02)	0.360
	Model A			
		ΔGNRI (per 1-point increase)	0.96 (0.94–0.99)	0.008
		ECOG PS (1 vs 0)	1.37 (0.89–2.11)	0.143
		Regimen (mFOLFIRINOX vs GnP)	1.25 (0.77–2.02)	0.351
		Ascites (Present vs Absent)	1.33 (0.82–2.14)	0.233
	Model B			
		ΔGNRI (per 1-point increase)	0.96 (0.94–0.99)	0.008
		ECOG PS (1 vs 0)	1.37 (0.89–2.11)	0.143
		Regimen (mFOLFIRINOX vs GnP)	1.25 (0.77–2.02)	0.351
		Distant metastasis (Present vs Absent)	1.33 (0.83–2.15)	0.233
	Model C			
		ΔGNRI (per 1-point increase)	0.96 (0.94–0.99)	0.016
		ECOG PS (1 vs 0)	1.47 (0.99–2.22)	0.072
		Regimen (mFOLFIRINOX vs GnP)	1.28 (0.77–2.08)	0.315
		Total bilirubin (per 1mg/dL increase)	0.98 (0.95–1.02)	0.565
	Model D			
		ΔGNRI (per 1-point increase)	0.96 (0.94–0.99)	0.012
		Regimen (mFOLFIRINOX vs GnP)	1.26 (0.61–2.59)	0.524
		ΔGNRI×Regimen interaction	1.01 (0.94–1.09)	0.672

Footnotes:

ΔGNRI was analyzed as a continuous variable (per 1-point increase). Model A additionally adjusted for ascites. Model B additionally adjusted for distant metastasis. Model C additionally adjusted for total bilirubin. Model D included an interaction term between ΔGNRI and chemotherapy regimen. Hazard ratios are shown with 95% confidence intervals.

A two-sided P value <0.05 was considered statistically significant. Abbreviations: CI, confidence interval; ECOG PS, Eastern Cooperative Oncology Group performance status; GNRI, Geriatric Nutritional Risk Index; ΔGNRI, change in Geriatric Nutritional Risk Index; HR, hazard ratio; OS, overall survival; TTF, time to treatment failure.

Supplementary Table S9. Exploratory Cox interaction analyses evaluating whether DTI duration modified the association between ΔGNRI and survival outcomes.

Endpoint	Interaction	HR (95% CI)	p Value
Time to treatment failure	ΔGNRI × Longer DTI	1.048 (0.989–1.111)	0.110
Overall survival	ΔGNRI × Longer DTI	1.011 (0.958–1.067)	0.695

Footnotes: DTI duration was dichotomized using the median value as the cut-off. ΔGNRI was analyzed as a continuous variable. The interaction term represents the interaction between continuous ΔGNRI and longer DTI duration in Cox proportional hazards models. Abbreviations: CI, confidence interval; DTI, diagnostic-to-treatment interval; GNRI, Geriatric Nutritional Risk Index; HR, hazard ratio; OS, overall survival; TTF, time to treatment failure; ΔGNRI, change in Geriatric Nutritional Risk Index.

Supplementary Table S10. Comparison of dynamic (ΔGNRI) and static pre-chemotherapy GNRI in multivariable models for early treatment discontinuation.

Comparison of ΔGNRI and static pre-chemotherapy GNRI (<92) in multivariable logistic regression models for early treatment discontinuation.

Variable	OR (95% CI)	p Value
Model A		
ΔGNRI (per 1-point increase)	0.89 (0.83–0.95)	0.001
ECOG PS (1 vs 0)	3.48 (1.31–9.22)	0.012
Model B		
Pre-chemotherapy GNRI (<92 vs ≥92)	4.91 (1.53–15.7)	0.007
ECOG PS (1 vs 0)	2.62 (1.01–6.81)	0.048
Model C		
ΔGNRI (per 1-point increase)	0.91 (0.84–0.99)	0.037
ECOG PS (1 vs 0)	2.94 (1.08–7.98)	0.034
Pre-chemotherapy GNRI (<92 vs ≥92)	2.55 (0.67–9.67)	0.169

Footnotes:

GNRI <92 was used as a predefined threshold for moderate-to-severe nutritional risk. Model A included ΔGNRI and ECOG PS. Model B included static pre-chemotherapy GNRI (<92) and ECOG PS. Model C simultaneously included ΔGNRI, static GNRI, and ECOG PS. Odds ratios are presented with 95% confidence intervals. A two-sided *p* value <0.05 was considered statistically significant. Abbreviations: CI, confidence interval; ECOG PS, Eastern Cooperative Oncology Group performance status; GNRI, Geriatric Nutritional Risk Index; ΔGNRI, change in Geriatric Nutritional Risk Index; OR, odds ratio.

Supplementary Table S11. Sensitivity analyses incorporating ΔGNRI, ΔNLR, and total bilirubin for early treatment discontinuation.

Multivariable logistic regression models evaluating the association between ΔGNRI and early treatment discontinuation after incorporating changes in NLR (ΔNLR) and total bilirubin.

Variable	OR (95% CI)	p Value
Model A		
Regimen (mFOLFIRINOX vs GnP)	3.54 (1.04–12.10)	0.043
ECOG PS (1 vs 0)	5.00 (1.68–14.80)	0.003
ΔGNRI (per 1-point increase)	0.89 (0.83–0.95)	0.001
Model B		
Regimen (mFOLFIRINOX vs GnP)	3.26 (0.99–10.70)	0.051
ECOG PS (1 vs 0)	4.86 (1.72–13.80)	0.002
ΔNLR (per 1-point increase)	1.14 (0.99–1.32)	0.057
Model C		
Regimen (mFOLFIRINOX vs GnP)	3.59 (1.04–12.40)	0.043
ECOG PS (1 vs 0)	5.20 (1.72–15.70)	0.003
ΔGNRI (per 1-point increase)	0.89 (0.83–0.96)	0.003
ΔNLR (per 1-point increase)	1.13 (0.96–1.26)	0.145
Model D		
ECOG PS (1 vs 0)	5.52 (1.77–17.20)	0.003
Regimen (mFOLFIRINOX vs GnP)	3.46 (0.98–12.30)	0.054
ΔGNRI (per 1-point increase)	0.89 (0.83–0.96)	0.002
ΔNLR (per 1-point increase)	1.13 (0.98–1.30)	0.097
Total bilirubin (per 1mg/dL increase)	1.05 (0.97–1.14)	0.244

Footnotes:

Odds ratios (ORs) are presented per 1-unit increase for continuous variables. All models adjusted for chemotherapy regimens and ECOG performance status. Model A additionally evaluated ΔGNRI. Model B additionally evaluated ΔNLR. Model C included both ΔGNRI and ΔNLR. Model D further adjusted for total bilirubin. Early treatment discontinuation was defined as cessation of first-line chemotherapy before the first radiologic response evaluation. Odds ratios are shown with 95% confidence intervals. A two-sided *p* value <0.05 was considered statistically significant.

Abbreviations: CI, confidence interval; ECOG PS, Eastern Cooperative Oncology Group performance status; GNRI, Geriatric Nutritional Risk Index; ΔGNRI, change in Geriatric Nutritional Risk Index; ΔNLR, change in neutrophil-to-lymphocyte ratio; OR, odds ratio.
